# Atherogenesis in Psoriasis: Evaluation of the Serum Activities of Non-high-density Lipoprotein Cholesterol and Other Lipids Among Newly Diagnosed Psoriasis Patients

**DOI:** 10.7759/cureus.4203

**Published:** 2019-03-07

**Authors:** Sudhakar Thungaturthi, Sabitha Vadakedath, Prathyusha Pavuluri, Jhansi Rani, Rajkumar Gundu, Jai Bheem, Venkataramana Kandi

**Affiliations:** 1 Biochemistry, Chalmeda Anandrao Institute of Medical Sciences, Karimnagar, IND; 2 Biochemistry, RVM Institute of Medical Sciences and Research Center, Hyderabad, IND; 3 Microbiology, Prathima Institute of Medical Sciences, Karimnagar, IND

**Keywords:** atherogenesis, psoriasis, non-hdl-c, lipids, total cholersterol, triglycerides, cardiovascular risk, inflammatory disease, cardiovascular disease

## Abstract

Introduction

Psoriasis is a chronic inflammatory skin disorder which commonly affects people aged between 15-25 years with a 2-3 % prevalence rate throughout the world. Psoriasis is a systemic inflammatory disease associated with severe co-morbidities that include cardiovascular risk. Although changes in the atherogenic lipids among psoriasis patients is already documented, very little is known about their role in atherogenesis among the new onset cases of psoriasis. Hence, this study is undertaken to assess the activities of non-high-density lipoprotein cholesterol (non-HDL-C) and other lipids among newly diagnosed psoriasis patients.

Methods

The study included 25 new onset cases of psoriasis patients aged between 20-60 years (mean age 38.2 years) attending the Dermatology outpatient department (OPD) of the Chalmeda Anandrao Institute of Medical Sciences (CAIMS), Karimnagar, Telangana, India, a tertiary care teaching hospital. An equal number of healthy individuals were included as controls. Blood was collected from all the subjects included in the study and was analyzed for various lipid parameters that included total cholesterol (TC), HDL-C, and triglycerides. The non-HDL-C and low-density lipoprotein cholesterol (LDL-C) were later calculated manually by using the standard formulae. The data were tabulated using Microsoft Excel and was analyzed for their statistical significance using the Student t-test.

Results

The results demonstrated a statistically significant difference in the lipid parameters between the cases and controls. Among the parameters measured, the pro-atherogenic lipids including the LDL-C and non-HDL-C activities among the cases (LDL-C 171.46±17.13, p=0.0002; non-HDL-C 213.27±20.17, p ≤ 0.0001) and controls (LDL-C 91.04±11.41, p=0.0002; Non-HDL-C 119.0± 12.28, p ≤ 0.0001) were found to be statistically significant. The ratios of non-HDL-C to HDL-C and total cholesterol to HDL-C both among the cases (7.10±0.1, 8.13±1.2) and control groups (3.05±0.30, 4.03±0.42) were also showing a statistically significant difference.

Conclusion

The results clearly demonstrate the significance of the evaluation of lipids among newly diagnosed cases of psoriasis patients. The activities of different lipoproteins including the non-HDL-C and LDL-C revealed an increase among the psoriasis patients. The ratios of non-HDL-C to HDL-C and TC to HDL-C also showed significant variability. Further, to establish their clinical utility in the development of cardiovascular disease (CVD), and to manage appropriately, a regular follow-up of such parameters both before and after initiation of treatment is required.

## Introduction

Psoriasis is a chronic, systemic inflammatory disease characterized by the development of patchy lesions on the skin. The lesions are reddish (sometimes silvery), dry, scaly, and vary in the size and could be localized or involve skin throughout the body correlating with the intensity of the disease. The disease severity is measured by the psoriasis area severity index (PASI), by measuring the surface area of the skin involved and grading accordingly (0-no disease and 72-severe disease). Psoriasis is associated with several comorbidities, including the risk of cardiovascular disease (CVD) [1-2]. Based on the type of clinical presentation, psoriasis may be classified as plaque/scaly, guttae, inverse, pustular, erythrodermic, and arthritic psoriasis. Among these, the plaque psoriasis is found to be the most prevalent one. Psoriasis may be associated with dyslipidemia and immunological abnormalities and therefore could be called as an immuno-metabolic disease. Psoriasis has been associated with the atherogenic lipid profile at a later stage in the disease [3-4]. The lipid abnormalities among the chronic psoriasis patients were used to predict CVD and the mortality [5-6]. 

Although the low-density lipoprotein cholesterol (LDL-C) is predominantly used as a predictor of atherogenesis and CVD, previous studies have highlighted the significance of non-high-density lipoprotein cholesterol (non-HDL-C) which represents the cholesterol content present in all lipoproteins (LDL-C, very LDL-C (VLDL-C), intermediate-density lipoprotein cholesterol (IDL-C)) as a true indicator of atherogenesis [7-8]. The effectiveness of non-HDL-C over the LDL-C in predicting the CVD was recently reported. It was also suggested that the non-HDL-C levels must be added to the lipid profile while predicting CVD [9]. The Incremental Disease in Endpoints through Aggressive Lipid-lowering (IDEAL) trial studies have also reported the activities of non-HDL-C and Apolipoprotein B (Apo B) in predicting CVD related mortality [10]. 

Epidemiological studies have previously confirmed the superiority of non-HDL-C over LDL-C in predicting CVD and the resultant morbidity and mortality [11-12]. The association of non-HDL-C estimation in predicting other lipid abnormalities and the resultant morbidity among psoriasis patients has been reported previously [13]. 

Since not many studies have estimated the non-HDL-C and other lipids among newly diagnosed cases of psoriasis, the present study aims to evaluate the activities of non-HDL-C and other lipids in newly diagnosed patients of psoriasis.

## Materials and methods

The present study included subjects selected from the patients attending the outpatient department (OPD) of Dermatology attached to the Chalmeda Anandrao Institute of Medical Sciences (CAIMS), Karimnagar, Telangana, India. This prospective case-control study was conducted from January 2018 to June 2018. A total of 25 patients aged between 20-60 years (mean age 38.2 years) diagnosed with psoriasis (16 males and nine females) were included as cases and an equal number of sex and age-matched healthy subjects were included as controls. An informed and oral consent was obtained from all the participants and the study was approved by the institutional ethical committee. 

All patients who presented/diagnosed recently with psoriasis and those who were not using any topical or systemic drugs were included in the study. Patients with a history of other metabolic disorders, alcoholism, smokers, pregnant women, women taking oral contraceptives, post-menopause aged women and those on lipid-lowering drugs were excluded from the study.

**Figure 1:**
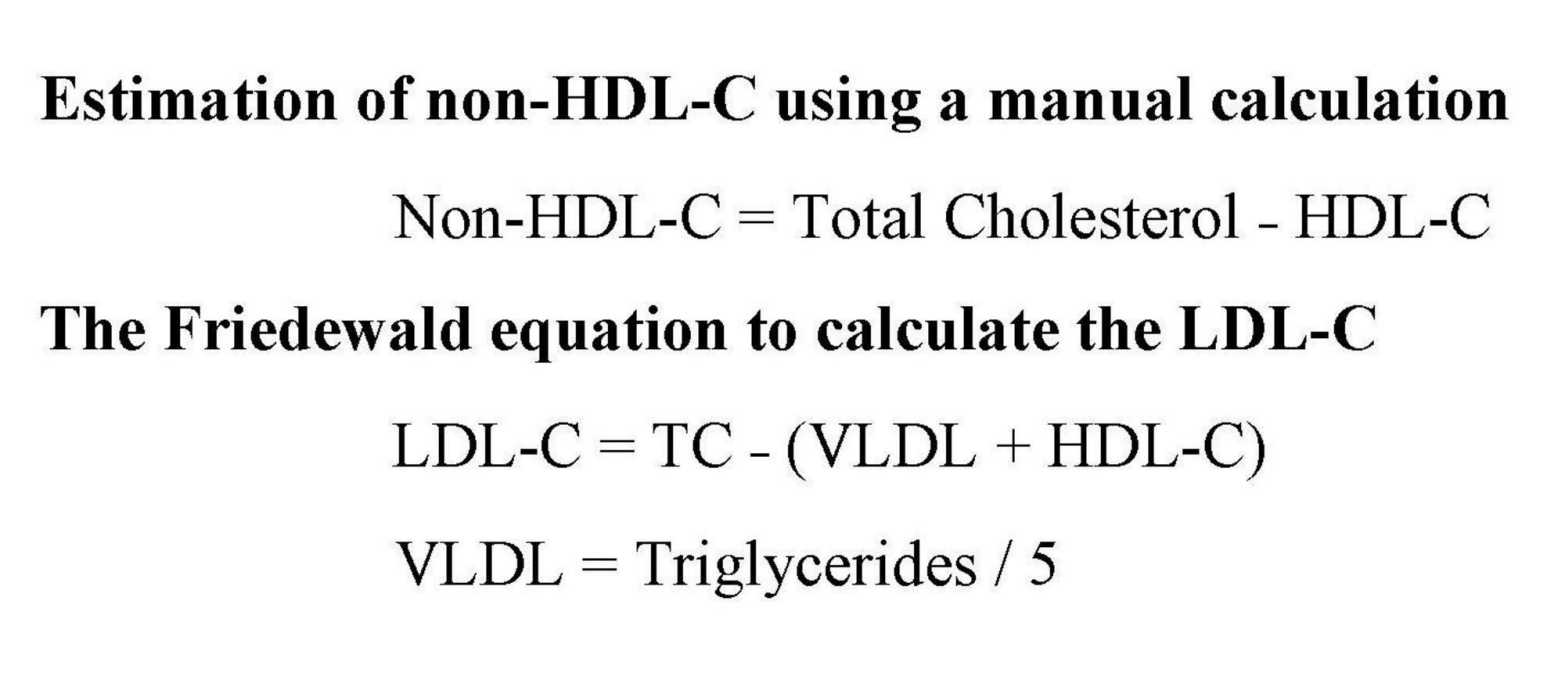
Estimation of non-HDL-C and LDL-C using standard formulas Non-HDL-C: Non-high-density lipoprotein cholesterol; HDL-C: High-density lipoprotein cholesterol; LDL-C: Low-density lipoprotein cholesterol; TC: Total cholesterol; VLDL: Very low-density lipoproteins

Five milliliters of venous blood was drawn aseptically from all the study subjects. The biochemical parameters analyzed included were serum total cholesterol (TC), HDL-C, and triglycerides. A semi-automated analyzer using the DiaSys kits was used for the estimation. The LDL-C was calculated using the Friedewald formula and non-HDL-C was calculated manually as shown in Figure 1 [14]. 

## Results

The results demonstrated a statistically significant difference in the lipid parameters between the cases (TC 245± 18.80, p ≤ 0.0001; LDL-C 171.46±17.13, p=0.0002; HDL-C 30.17±2.65, p ≤ 0.0001; Non-HDL-C 213.27±20.17, p ≤ 0.0001 and serum triglycerides 208.92± 17.29, p ≤ 0.0001) and controls (TC 157.64±13.02, p ≤ 0.0001; LDL-C 91.04±11.41, p=0.0002; HDL-C 39.08± 3.08, p ≤ 0.0001; Non-HDL-C 119.0± 12.28, p ≤ 0.0001 and serum triglycerides 137.64± 20.47; p ≤ 0.0001). The ratios of non-HDL-C to HDL-C and TC to HDL-C both among the cases (7.10±0.1, 8.13±1.2) and control (3.05±0.30, 4.03±0.42) groups were noted to be showing a statistical difference. The mean and standard deviation (SD) values for TC, HDL-C, non-HDL-C, and triglycerides among the psoriasis group and the control group is shown in Table 1.

**Table 1:** The comparison of lipid parameters among the psoriasis and the control group HDL-C: High-density lipoprotein cholesterol; Non-HDL-C: Non-high-density lipoprotein cholesterol; LDL-C: Low-density lipoprotein cholesterol; TC: Total Cholesterol

Parameters	Control group (Mean±SD)	Psoriasis group (Mean±SD)	p-value
TC	157.64±13.02	245±18.80	≤ 0.0001
LDL-C	91.04±11.41	171.46±17.13	=0.0002
HDL-C	39.08±3.08	30.17±2.65	≤ 0.0001
Non-HDL-C	119.0± 12.28	213.27±20.17	≤ 0.0001
Triglycerides	137.64±20.47	208.92±17.29	≤ 0.0001

The mean values for the ratios of non-HDL-C to HDL-C and TC to HDL-C both among the cases and control groups are shown in Table 2. 

**Table 2:** The ratio of non-HDL-C to HDL-C and total cholesterol to HDL-C in controls and the psoriasis groups Non-HDL-C: Non-high-density lipoprotein cholesterol; HDL-C: High-density lipoprotein cholesterol; TC: Total cholesterol

Parameter	Control group (Mean±SD)	Psoriasis group (Mean±SD)	p-value
Non-HDL-C/HDL-C	3.05±0.30	7.10 ±0.1	≤ 0.0001
TC/HDL-C	4.03±0.42	8.13±1.2	≤ 0.0001

## Discussion

**Figure 2:**
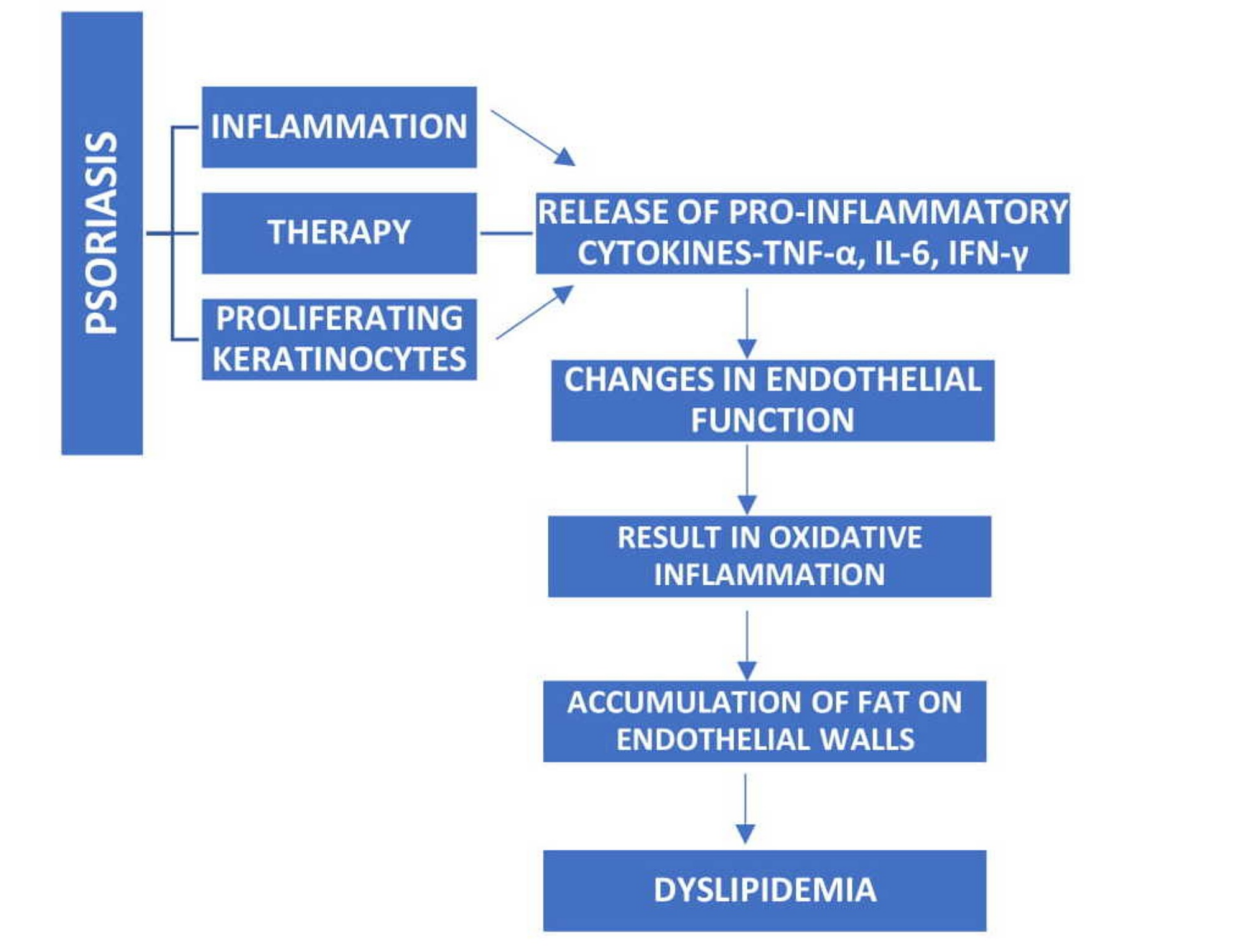
Mechanism of atherogenesis among psoriasis patients TNF-α: Tumor necrosis factor-α; IL-6: Interleukin-6; IFN-γ: Interferon gamma

Psoriasis is a chronic inflammatory condition of skin due to a hyperactive immune system with a genetic predisposition. During the early stages of psoriasis, the keratinocyte related skin changes are seen. But, during the later stages, the immunological changes in the cells could complicate the disease course. The management of psoriasis includes the use of corticosteroids, retinoids, and cyclosporin, which could further aggravate inflammation by suppressing the immunity and trigger dyslipidemia in psoriasis as shown in Figure 2.

Activated T lymphocytes i.e., T helper cells 1 (Th1) and Th17) leads to a sequential release of pro-inflammatory molecules which may cause insulin resistance, dyslipidemia, atherogenesis, and CVDs. Zaba et al. showed that psoriatic dermal dendritic cells could induce T cell proliferation and also polarize T cells to generate Th17 and Th1 cells [15].

During physical trauma or microbial infection, the T cells may get stimulated to differentiate into Th17 cells in the presence of transforming growth factor beta (TGF-β) and interleukin 6 (IL- 6). Th17 cells, cytokines, and activated keratinocytes interact to cause an activated inflammatory network leading to clinical features of psoriasis [16-17]. The inflammatory metabolic changes, especially dyslipidemia (a disorder of lipoprotein metabolism) plays an important role in the development of cardiovascular disease in psoriasis patients [18-19]. Chronic inflammation followed by secretion of pro-inflammatory cytokines like tumor necrosis factor-α (TNF-α), IL6, interferon gamma (IFN-γ) etc., modulates the activity of lipoprotein lipase enzyme on the endothelial wall causing accumulation of oxidized LDL increasing the risk for CVDs [20-21]. Dyslipidemia among psoriatic patients may be due to loss of cholesterol from the body due to the exfoliation of the skin, and due to structural and functional changes in the intestine, as it plays an important role in absorption, and degradation of lipoproteins [22-23].

Atherogenesis (deposition of fat along the walls of blood vessels) is the major risk factor for heart diseases and among the psoriasis patients, it is a major cause of morbidity. Routinely, LDL-C activities are used to predict atherogenic risk among individuals. It was previously observed that the activities of LDL-C were normal in people with abdominal obesity, metabolic syndrome, and diabetic lipid disorders, but had increased activities of other atherogenic lipoproteins like the VLDL, IDL, and small dense LDL (Lp(a)). Hence measuring other lipid parameters which reflect atherogenic risk like the non-HDL could be useful. Thus, as an alternative, the activities of non-HDL-C may be used to predict pro-atherogenesis [24].

The non-HDL-C was found to be a better marker of atherogenesis than LDL-C in both primary and secondary prevention of CVD. Also, the estimation of non-HDL-C requires no fasting sample as compared to LDL-C as noted form earlier studies [25]. The World Health Organization (WHO) study, the Helsinki heart study, and other studies also recommend the use of non-HDL-C to predict the risk of atherogenesis [8-9, 26-27]

The activities of TC and non-HDL-C were found to correlate with the risk of atherogenesis and, therefore, it was suggested that non-HDL-C could be used as an alternate marker to assess the risk of CVD [28].

In the National Cholesterol Education Programme (NCEP), Adult Treatment Panel III (ATP III) guideline, the lowering of non-HDL-C is advised as a secondary goal when serum triglycerides were more than 200 mg/dl. Also, the target goal is always taken as 30 mg/dl higher than the LDL-C target for each NCEP risk category (≤ 100 mg/dl and ≤ 130 mg/dl ) [14]. The lipid-lowering therapy for non-HDL-C is stated to be a holistic principle in the management of dyslipidemia as compared to LDL-C activities. Therefore, the non-HDL-C is an ideal parameter in predicting dyslipidemia and atherogenesis among psoriasis patients as evidenced by the results of our study.

In the present study, the activities of different lipoproteins including the non-HDL-C and LDL-C revealed an increase among the psoriasis patients. The ratios of non-HDL-C to HDL-C and TC to HDL-C also showed significant variability. Further, to establish their clinical utility in the development of CVD, a regular follow-up of such parameters both before and after initiation of treatment is the need of the hour.

## Conclusions

Dyslipidemia in psoriasis patients appears to be multi-factorial. The study results clearly demonstrate an increase in the activities of pro-atherogenic lipoproteins. Activities of non-HDL-C, and LDL-C both were found to significantly increase among the cases. Since all the psoriasis patients recruited in the present study were newly diagnosed and that they were treatment naive, a regular follow-up of lipid parameters in these patients after the initiation of the therapy could increase the understanding of their potential role in atherogenesis and CVD related morbidity and mortality.
